# The Prevalence and Associated Factors of Domestic Violence Against Pregnant Women During the COVID‐19 Pandemic: A Systematic Review and Meta‐Analysis

**DOI:** 10.1002/brb3.70345

**Published:** 2025-02-28

**Authors:** Kosar Miraei Mohammadi, Zohreh Shahhosseini, Mahtab Haji Mohammadreza, Fatemeh Heshmatnia, Roya Nikbakht, Erfan Ghasemi, Maryam Jafari, Hamed Milani, Marzieh Azizi

**Affiliations:** ^1^ Student Research Committee Mazandaran University of Medical Sciences Sari Iran; ^2^ Department of Midwifery and Reproductive Health, Sexual and Reproductive Health Research Center Mazandaran University of Medical Sciences Sari Iran; ^3^ Department of Nursing and Midwifery, Semnan Branch Islamic Azad University Semnan Iran; ^4^ Department of Biostatics and Epidemiology, Faculty of Health Mazandaran University of Medical Sciences Sari Iran; ^5^ Non‐Communicable Diseases Research Center, Endocrinology and Metabolism Population Sciences Institute Tehran University of Medical Sciences Tehran Iran; ^6^ Department of Midwifery, School of Nursing and Midwifery Shiraz University of Medical Sciences Shiraz Iran; ^7^ Faculty of Medicine Mazandaran University of Medical Sciences Sari Iran

**Keywords:** COVID‐19 | domestic violence | physical | pregnancy | psychological | sexual

## Abstract

**Introduction:**

Domestic violence (DV) against women has been reported increasingly, especially during the pandemic worldwide. Exposure to DV during pregnancy is associated with various maternal and neonatal adverse consequences. Therefore, the current study aims to systematically investigate and analyze the prevalence and associated factors of DV or intimate partner violence (IPV) against pregnant women during the COVID‐19 pandemic.

**Methods:**

In this systematic review and meta‐analysis, systematic literature searches in electronic databases, including Google Scholar, PubMed, Web of Science, Scopus, ScienceDirect, and Scientific Information Database, were conducted from December 2023 to May 2024. Quality assessment of the included studies was performed using the Newcastle–Ottawa Scale for cross‐sectional and cohort studies. All included studies were entered into a meta‐analysis. The binomial distribution formula was used to calculate the variance of point prevalence. In addition, meta‐regression was used to assess the prevalence of DV based on the sampling place and quality of the included studies. All statistical analyses were performed with Stata version 11.0, Texas, USA.

**Results:**

Of 16 studies included, 156,775 pregnant women participated, and the sample sizes varied from 215 to 77,310 individuals. According to the combining the results of 12 studies, the overall prevalence of physical, psychological, and sexual violence against pregnant women during COVID‐19 was estimated at 13.83 (95% CI, 5.92%–21.73%), 40.02% (95% CI, 22.74%–57.30%), and 15.09% (95% CI, 6.49%–23.69%), respectively. The pooled prevalence of the total IPV against pregnant women during COVID‐19, according to the combined results of 15 studies, was estimated at 36.82% (95% CI, 22.24%–51.40%).

**Conclusion:**

Although the prevalence of all types of violence against pregnant women increased during the COVID‐19 pandemic compared to other times, the results of the present study indicated that psychological violence was the most common violence reported by pregnant women. Therefore, it seems that identifying high‐risk pregnant women as victims of violence is imperative to develop preventive interventions for this vulnerable group globally.

## Graphical abstract

GRAPHICAL ABSTRACT According to the combining the results of 12 studies, the overall prevalence of physical, psychological, and sexual violence against pregnant women during COVID‐19 was estimated at 13.83 (95% CI, 5.92%–21.73%), 40.02% (95% CI, 22.74%–57.30%), and 15.09% (95% CI, 6.49%–23.69%), respectively. The pooled prevalence of the total IPV against pregnant women during COVID‐19, according to the combined results of 15 studies, was estimated at 36.82% (95% CI, 22.24%–51.40%). The risk factors of all types of violence during pregnancy were shown in the graphical abstract.

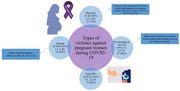

## 1 Introduction

Domestic violence (DV) against women has been reported increasingly, especially during the pandemic worldwide (Piquero et al. 2021). DV against women is considered a significant public health problem and may include physical, sexual, psychological, verbal, or emotional abuse (Wu et al. 2022). Many women are abused by their partners, which may be continued or become worse during pregnancy (ACOG). In this regard, clinical‐based studies showed an increase in intimate partner violence (IPV) during pregnancy, but pregnancy is not a risk factor for IPV (Alemu et al. 2024; Teshome et al. 2021). It is estimated that more than 30% of women experience IPV lifetime, and also 3%–9% of women are exposed to IPV during pregnancy (Muldoon et al. 2021). The results of a study indicated that pregnant women were 2.7–3.9 times more likely to experience physical and twice more likely to be exposed to sexual violence compared to nonpregnant women (Brownridge et al. 2011).

Even though there is limited evidence regarding the adverse complications of pregnancy associated with the COVID‐19 pandemic, the physiological relative suppression of the immune system related to pregnancy and also cardiopulmonary changes lead to pregnant women being more susceptible to infectious respiratory diseases (Brownridge et al. 2011). Therefore, fear of infection due to the COVID‐19 pandemic and its possible negative effect on pregnancy outcomes leads to considerable anxiety and stress among pregnant women and their families (Fetene et al. 2022) and increased physical distancing and isolation due to the pandemic among pregnant women and their husbands (Wu et al. 2022).

The World Health Organization (WHO) and the Centers for Disease Control and Prevention released a report that the COVID‐19 pandemic restrictions led to increased cases of violence against women, so one in four women reported that they experienced each type of IPV during the pandemic (Fetene et al. 2022). The literature review showed that during COVID‐19, the prevalence of DV has risen dramatically in many countries (Graham‐Harrison et al. 2020; Teshome et al. 2021). In other words, the coronavirus disease 2019 leads to increased severity of IPV in women with a history of each type of abuse (Piquero et al. 2021).

Exposure to DV during pregnancy is associated with various maternal and neonatal adverse consequences (Fetene et al. 2022). These adverse outcomes included injuries and chronic pain, sexually transmitted diseases, pelvic inflammatory diseases, and adverse pregnancy outcomes such as poor weight gain, abortion, low birth weight, preterm labor, cesarean delivery, and early neonatal death (Garg et al. 2020; Islam et al. 2017; Muldoon et al. 2021). In addition, DV is associated with increased negative psychological consequences, including anxiety, stress, depression, post‐traumatic stress disorder, suicidal attempts, irregular prenatal care attendance, and drug and alcohol abuse (Belay et al. 2019; Islam et al. 2017).

Pregnant women and their husbands need access to timely antenatal care and support during pregnancy, especially during the COVID‐19 pandemic (Meaney et al. 2022). During the pandemic, as healthcare professionals are at high risk of COVID‐19 infection, there may be fewer healthcare professionals available to provide antenatal care to pregnant women (Wilson et al. 2021). Research showed that nonattendance of pregnant women results in increased adverse pregnancy outcomes such as undetected intrauterine growth restriction, preterm birth, and perinatal mortality (Heaman et al. 2019; Meaney et al. 2022).

Although there are some studies regarding the prevalence of DV or IPV against pregnant women during the COVID‐19 pandemic (Aghababaei et al. 2024; Huldani et al. 2022; Raziani et al. 2024), these studies assessed studies until 2022, and also the associated factors of DV or IPV were not evaluated in these studies, so this study aimed to systematically investigate and analyze the prevalence and associated factors of DV or IPV against pregnant women during the COVID‐19 pandemic.

## 2 Methods

### 2.1 Study Design and Protocol Registration

This systematic review and meta‐analysis were conducted according to the Preferred Reporting Items for Systematic Reviews and Meta‐Analyses (PRISMA) statements 2020 (Page et al. 2021). Before conducting this study, its protocol was registered in the International Prospective Register of Systematic Reviews (PROSPERO; Registration no. CRD42024502071) and is available from https://www.crd.york.ac.uk/prospero/.

The PECO (participant, exposure, comparison, and outcome) model was used to find eligible study criteria; the participants were pregnant women who had experienced DV during pregnancy during the COVID‐19 pandemic. The types of “exposure” covered were each type of violence; the “comparison” was not applicable, and the “outcome” was the prevalence of DV and associated factors during pregnancy.

#### 2.1.1 Literature Search and Search Strategy

A comprehensive systematic literature search in electronic databases, including Google Scholar, PubMed, Web of Science, Scopus, ScienceDirect, and Scientific Information Database, was conducted to collect the data for this systematic review. The search interval was from December 2023 to May 2024 and was performed by two authors (M.A. and F.H.). To search in the mentioned databases, first, Medical Subject headings were used to extract search terms, including: (“cross‐sectional studies” OR “descriptive studies” OR “prospective study” OR “cohort”] AND (“domestic violence” OR “marital violence” OR “physical violence” OR “psychological violence” OR “sexual violence” OR “intimate partner violence” [MeSH]) AND (“pregnancy” OR “pregnant women” OR “perinatal” OR “prenatal” [MeSH] OR “antenatal”) AND (“COVID‐19” OR “coronavirus” OR “SARS‐COV‐2” [MeSH]). We searched other databases according to the specific search guidelines for advanced searches provided by each database. The details of the search strategy are shown in Table 1. The authors searched the reference lists of included studies to confirm that all relevant records had been identified. The reference manager software EndNote X8 was used to manage the references.

**TABLE 1:** The search strategy in databases.

**Last search date**	**The latest search was performed from “October 2023 to May 2024”**		
**Database**	**Search strategy**	**Number of extracted studies**	**Search filters**
PubMed	(Domestic violence) OR (intimate partner violence) OR (partner violence) OR (physical violence) OR (emotional violence) OR (psychological violence) OR (sexual violence) AND (pregnancy) OR (pregnant women) AND (COVID‐19) OR COVID‐19 pandemic)	1385	Text availability = Free full text – Article attribute: Associated data – Article type: Observational study – Species: Human Publication date: 2020–2024
Scopus	TITLE‐ABS‐KEY (Domestic violence OR intimate partner violence OR partner violence OR physical violence OR sexual violence OR emotional violence) AND (pregnant OR pregnancy OR perinatal OR prenatal) AND (COVID‐19) OR (COVID‐19 pandemic)	289	– Subject areas: Medicine – Document types: Article – Source type: Journal
WOS	Domestic violence OR intimate partner violence OR partner violence OR physical violence OR sexual violence OR emotional violence) AND (pregnant OR pregnancy OR perinatal OR prenatal) AND (COVID‐19) OR (COVID‐19 pandemic)	1077	– Publication year: 2020–2024 – Document type: Article – Language: English – Open access: Free to read
ScienceDirect	(Domestic violence OR intimate partner violence OR physical violence OR psychological violence OR sexual violence AND pregnant women AND COVID‐19)	729	– Article type: Research article
SID	Domestic violence OR intimate partner violence AND pregnant women AND COVID‐19	32	Year of publication: 2020–2024
Google Scholar	(“Domestic violence” OR “violence” OR “intimate partner violence”) AND (“pregnancy” OR “pregnant women”) AND (COVID‐19)	3520	– Year of publication: 2020–2024 – Sort by relevance – Keywords in the title of the article

#### 2.1.2 Inclusion and Exclusion Criteria

All free full‐text cohort and cross‐sectional studies with various types of sampling methods (i.e., random and nonrandom methods) for assessing the prevalence of DV or IPV (physical, psychological, and sexual violence) or total IPV (%) against pregnant women since the outbreak of the COVID‐19 pandemic were included.

Case reports, case series, letters, brief communications, randomized controlled trials (RCT), quasi‐experimental review studies, and conference presentations regarding DV against pregnant women or DV against women or men during the COVID‐19 pandemic, and also gray literature, were excluded. In addition, studies regarding violence against pregnant women that did not report the prevalence of DV or IPV or were conducted in a non‐COVID‐19 era were also excluded.

#### 2.1.3 Type of Outcome Assessment

On the basis of the relevant published articles, the outcome measured was the meta‐analysis of the prevalence of DV or IPV, such as physical, psychological, and sexual violence, and also total IPV against pregnant women during the COVID‐19 pandemic. DV OR IPV was defined as physical, psychological, and sexual abuse or any threatening behaviors that lead to adverse outcomes.

Physical violence refers to an act attempting to or resulting in pain or physical injury and includes beating, burning, punching, biting, or tearing out hair (Loiseau et al. 2021). Psychological violence is a common form of gender‐based violence that includes any intentional course of conduct that seriously impairs another person's psychological integrity through coercion or threats (Every‐Palmer et al. 2020). Sexual violence in this research refers to any harmful or unwanted sexual act or sexual act through violence or coercion or an act against a person's sexuality without their consent (Sifat 2020). DV in this study refers to each type of violence such as physical, sexual, emotional, psychological, or threats of actions or other patterns of coercive behavior that influence another person within an intimate partner relationship (Boxall, Morgan, and Brown 2020). IPV refers to physical, sexual, or psychological abuse of a person by their partner or spouse (Rahman et al. 2022).

#### 2.1.4 Data Collection and Extraction

Two authors (M.A. and K.M.M.) independently reviewed the collected articles and extracted the data using a uniform standardized data collection form. If a study was relevant, the full text was reviewed for further assessment. Any discrepancies were resolved through discussion with a third author (Z.S.) during the search and screening procedure. The extracted variables included the first author's name, publication year, country, study design, sample size, mean ± standard deviation (SD) of participants’ age (in years), sampling place, sampling method, measurement tools, the prevalence of each type of DV or IPV, prevalence of total IPV, and associated factors of DV or IPV during pregnancy (Table 2).

**TABLE 2:** The characteristics of the included studies (*n* = 17).

Reference	Study design	Sampling size	Age of participants	Sampling place	Sampling method	Measurement tools	Prevalence of each type of DV or IPV (%)				Associated factors of DV or IPV
							Physical	Psychological	Sexual	Total IPV	
Biagio et al. (2024), Brazil	Cohort	400	20–30	Maternity clinic	Convenience	DVQST	—	19.5	—	52.2	Any violence was associated with being single (OR = 2.95, 95% CI, 1.34%–5.76%), parity 0 (OR = 3.19, 95% CI, 1.38%–7.39%), and parity ≥3 (OR = 3.54, 95% CI, 1.45%–8.66%) and mental health changes according to the GHQ (OR = 4.67, 95% CI, 2.90%–6.34%). Psychological violence was associated with single/divorced and widow (OR = 4.92, 95% CI, 2.80%–8.89%) and mental health changes according to the GHQ (OR = 5.93, 95% CI, 2.78%–9.10%)
Abujilban et al. (2023), Jordan	Cross‐sectional	232	Not reported	Maternity clinic	Convenience	DVQST	46.10	64.20	40.90	75.90	Lower educational level, weak mutual understanding, lower marriage duration, and employment status (housework) were associated with physical violence
Atilla et al. (2023), Turkey	Cross‐sectional	456	26.66 ± 5.45 and 30.63 ± 6.04	Hospital	Convenience	“Pregnant Information Form” and “Intimate Partner Violence” during pregnancy	6.6	33.3	5.7	44.1	Moderate‐bad‐very bad marital relationship (OR = 13.21, 95% CI, 6.29%–27.74%), partner unemployed (OR = 1.76, 95% CI, 1.00%–3.10%) and first pregnancy (OR = 1.68, 95% CI, 1.06%–2.65%) associated with IPV
Maharlouei et al. (2023), Iran	Cross‐sectional	830	<25 to >39	Maternity clinics	Mixed of randomized and convenience	Obstetric and medical history, IPV questionnaire	7.7	92.9	11.0	93.1	The 30–34 age group was a risk factor for physical DV Mother‐wanted pregnancy (OR = 0.26, 95% CI, 0.09%–0.79%) was protective of physical and father‐wanted pregnancy (OR = 0.20, 95% CI, 0.07%–0.56%) was a protective factor against sexual DV
Jalili et al. (2023), Iran	Cross‐sectional	308	18–43	Maternity clinics	Multi‐stage random	IPV questionnaire	14.9	74.4	17.2	19.2	Low educational status (*p* = 0.019), lower husband's education (*p* = 0.040), and worker husband's job (*p* = 0.017) were associated with IPV
Avalos et al. (2023), USA	Cross‐sectional	77,310	30.9 ± 5.3	Hospital	Electronic health records (convenience)	IPV questionnaire	—	—	—	1.58–3.17	An unsafe/unstable living situation (OR = 1.022, 95% CI, 1.016%–1.029%) was associated with IPV
Ozgurluk et al. (2023), Turkey	Cross‐sectional	68,356	28.8 ± 6.5	Hospital	Electronic health records (convenience)	IPV questionnaire	—	—	—	29.4–51.5	Not reported
Abujilban et al. (2022), Jordan	Cross‐sectional	215	28.6 ± 4.3	Online social media	Snow‐ball	DVQST	13.0	50.2	11.2	—	Marital conflict, verbal fighting, and weak understanding of each other (*p* < 0.05) were associated with IPV
Wu et al. (2022), China	Cross‐sectional	3434	<19 to >35	Hospital	Multistage random	Abuse Assessment Screen Questionnaire to evaluate IPV and General Anxiety Disorder Patient Health Questionnaire	0.6	2.2	0.7	2.2	Prenatal anxiety (OR = 4.20, 95% CI, 2.46%–7.16%) and depression (OR = 3.86, 95% CI, 2.09%–7.12%) were associated with IPV
Asratie (2022), Ethiopia	Cohort	774	31.3 ± 7.3	Hospital	Multistage systematic sampling	DV during pregnancy questionnaire	—	—	—	65.7	Unintended pregnancy (OR = 2.2; 95% CI, 1.0%–4.6%), no ambulance services (OR = 1.5, 95% CI, 1%–2.2%), not being the primary decision maker of family planning use (OR = 3.3; 95% CI, 1.6%–6.5%), not having the healthcare provider support (OR = 12; 95% CI, 6.3%–23.0%) were associated with DV
Fetene et al. (2022), Ethiopia	Cross‐sectional	590	15 to >30	Community (House to house collection)	Multistage random	EDHC, WHO‐MCVAW	29.8	26.8	22.2	39.2	Illiteracy (OR = 2.36, 95% CI, 1.33%–4.19%), having an illiterate husband (OR = 4.79, 95% CI, 2.69%–8.55%), decision‐making by only husband (OR = 4.91, 95% CI, 3.74%–9.33%) and economic downturns (OR = 9.03, 95% CI, 5.18%–15.98%) were associated with IPV
Teshome et al. (2021), Ethiopia	Cross‐sectional	464	28.1 ± 4.8	Maternity clinic	Not reported	Open data kit interview	30.3	72.7	48.5	7.1	Partner alcohol and drug use (OR = 3.36, 95% CI, 1.64%–6.91%), women's lost job (OR = 1.18, 95% CI, 0.39%–3.52%), husband's smoking (OR = 3.33, 95% CI, 0.36%–30.7%) and lower parity (OR = 1.93, 95% CI, 0.94%–3.94%) associated with IPV
Muldoon et al. (2021), Canada	Cross‐sectional	216	30–36	Hospital	Not reported	WHO‐MCVAW, EPDS	5.00	10.23	2.33	24.07	Household income below the municipal median (OR = 3.24, 95% CI, 1.87%–5.59%), nulliparity (OR = 1.18, 95% CI, 0.71%–1.97%), and postnatal depression (OR = 1.03, 95% CI, 1.00%–1.07%) were associated with perinatal IPV
Wood et al. (2022), Ethiopia	Cohort	2388	15–49	Maternity clinic	Purposive	10‐item RCTS	7.8	—	9.8	15.1	Urban residential place (OR = 2.09, 95% CI, 1.10%–3.96%) was associated with IPV
Naghizadeh et al. (2021), Iran	Cross‐sectional	250	30.57 ± 5.87	Hospital	Convenience	(WHO‐MCVAW), SF‐12 quality of life	4.8	32.8	12.4	35.2	Prolonged spouse's stay at home during the COVID‐19 (*p* = 0.020) was associated with DV
Krishnamurti et al. (2021), USA	Cross‐sectional	552	Not reported	Hospital	Convenience	Not reported	1.4	1.4	0.9	—	Not reported

Abbreviations: 10‐item RCTS, 10‐item Revised Conflict and Tactics Scale; DV, domestic violence; DVQST, Domestic Violence Questionnaire Screening Tool; EDHS, Ethiopia Demographic and Health Survey; EDPS, Edinburgh Postnatal Depression Survey; IPV, intimate partner violence; PDQ, Prenatal Distress Questionnaire; SCL‐90‐R, Symptom Checklist‐90; WHO‐MCVAW, World Health Organization Multi‐Country Violence Against Women.

#### 2.1.5 Methodological Quality Assessment (Risk of Bias Assessment)

Quality assessment of the study articles was performed using the Newcastle–Ottawa Scale (NOS) for cross‐sectional (Table 3) and cohort studies (Table 4). NOS is one of the most known scales for assessing quality and risk of bias in observational studies (Margulis et al. 2014; Stang 2010; G. A. Wells et al. 2000). In cross‐sectional studies, the NOS evaluates three quality parameters (selection, comparability, and outcome) divided across eight specific items; each item on the scale is scored from one point, except for the comparability parameter, which scores up to two points. Thus, the maximum for each study is 9, and studies with scores <6 points are identified as having a high risk of bias (Luchini et al. 2017; G. Wells et al. 2016).

**TABLE 3:** Methodological quality assessment through Newcastle–Ottawa scale (for cross‐sectional studies).

Row	First author/year	Selection representativeness of samples	Sample size	Nonrespondent	Ascertainment of exposure	Comparability assessment of outcomes	Statistical tests	Scores 0–10 stars
1	Abujilban et al. (2023), Jordan	*b* (*)	*a* (*)	*c*	*a* (**)	*c* (*)	*a* (*)	6
2	Atilla et al. (2023), Turkey	*b* (*)	*a* (*)	*c*	*a* (**)	*c* (*)	*a* (*)	6
3	Maharlouei et al. (2023), Iran	*a* (*)	*a* (*)	*c*	*a* (**)	*c* (*)	*a* (*)	6
4	Jalili et al. (2023), Iran	*a* (*)	*a* (*)	*c*	*a* (**)	*c* (*)	*a* (*)	6
5	Avalos et al. (2023), USA	*b* (*)	*a* (*)	*c*	*a* (**)	*c* (*)	*a* (*)	6
6	Ozgurluk et al. (2023), Turkey	*b* (*)	*a* (*)	*c*	*a* (**)	*c* (*)	*a* (*)	6
7	Abujilban et al. (2022), Jordan	*b* (*)	*a* (*)	*c*	*a* (**)	*c* (*)	*a* (*)	6
8	Wu et al. (2022), China	*a* (*)	*a* (*)	*c*	*a* (**)	*c* (*)	*a* (*)	6
9	Fetene et al. (2022), Ethiopia	*b* (*)	*a* (*)	*c*	*a* (**)	*c* (*)	*a* (*)	6
10	Teshome et al. (2021), Ethiopia	*d*	*a* (*)	*c*	*a* (**)	*c* (*)	*a* (*)	5
11	Muldoon et al. (2021), Canada	*d*	*a* (*)	*c*	*a* (**)	*c* (*)	*a* (*)	5
12	Wood et al. (2022), Ethiopia	*b* (*)	*a* (*)	*c*	*a* (**)	*c* (*)	*a* (*)	6
13	Krishnamurti et al. (2021), USA	*d*	*a* (*)	*c*	*a* (*)	*c* (*)	*a* (*)	4
14	Naghizadeh et al. (2021), Iran	*b* (*)	*a* (*)	*c*	*a* (**)	*c* (*)	*a* (*)	6

**TABLE 4:** Newcastle–Ottawa quality assessment scale (for Cohort studies).

Row	First author/year	Selection				Comparability	Outcome			Scoring
		**Representativeness of the sample**	**Selection of the nonexposed cohort**	**Ascertainment of exposure**	**Demonstration that outcome of interest was not present at the start of the study**	**The subjects in different outcome groups are comparable, based on the study design or analysis. Confounding factors are controlled**	**Assessment of the outcome**	**Was follow‐up long enough for outcomes to occur**	**Adequacy of follow‐up of cohorts**	
1	Biagio LD, (2024), Brazil (Biagio et al. 2024)	*b* (*)	*a* (*)	*b* (*)	*a* (*)	*a* (*)	*b* (*)	*b*	*d*	Good
2	Asratie (2022), Ethiopia (Asratie 2022)	*b* (*)	*a* (*)	*b* (*)	*a* (*)	*a* (*)	*b* (*)	*a* (*)	*a* (*)	Good

The NOS for cohort studies evaluates three quality parameters (selection, comparability, and outcome) divided across nine specific items; it slightly differs when scoring cross‐sectional, case‐control, and cohort studies. The scoring for cohort studies is as follows: good quality (3 or 4 stars in the selection domain, 1 or 2 stars in the comparability domain, and 2 or 3 stars in the outcome/exposure domain), fair quality (2 stars in selection domain, 1 or 2 stars in comparability domain, and 2 or 3 stars in outcome/exposure domain), and poor quality (0 or 1 star in selection domain, 0 stars in comparability domain, or 0 or 1 star in outcome/exposure domain) (Luchini et al. 2017; G. Wells et al. 2016; G. A. Wells et al. 2000).

### 2.2 Statistical Analysis

All included studies were entered into a meta‐analysis. The binomial distribution formula was used to calculate the variance of point prevalence. The heterogeneity among studies was tested using the *Q* statistic with a significance level of 0.1 and the evolution of heterogeneity with *I* squared (*I* ^2^). We applied the random effects model to estimate the prevalence after testing heterogeneity. The combined prevalence of DV was reported on the basis of the sampling methods by three categories (clinical, hospital, and other places) and quality score assessment (<6 and 6 or more according to the scoring of NOS for cross‐sectional studies). We used meta‐regression to assess the association of sampling place and quality assessment with the prevalence of each DV. All statistical analyses were performed with Stata version 11.0, Texas, USA.

## 3 Results

### 3.1 Search Results

**FIGURE 1:**
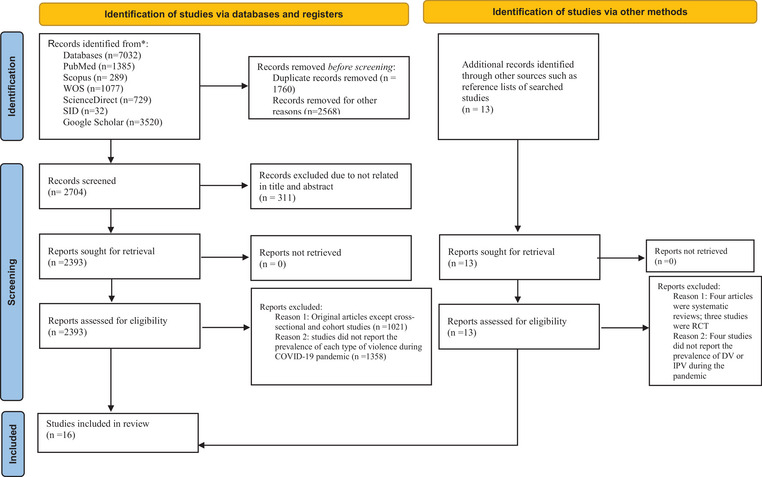
The PRISMA diagram for the search of records and study selection. PRISMA, Preferred Reporting Items for Systematic Reviews and Meta‐Analyses.

The systematic search resulted in 7032 articles; after removing the duplicates (*n* = 1760) and records removed for other reasons (*n* = 2568), 2704 studies remained. In this stage, 311 studies were excluded on the basis of the titles and abstracts. During the review of the full texts, a collection of articles was excluded if they were RCT, quasi‐experimental review studies, case reports, case series, letters, brief communications, and conference presentations regarding DV against pregnant women or DV against women or men during the COVID‐19 pandemic (*n* = 1021) and studies did not report the prevalence of each type of violence during the COVID‐19 pandemic (*n* = 1358). In addition, 13 articles were identified through reference lists of included studies. Among them, four articles were systematic reviews, three studies were RCT, and four studies did not report the prevalence of DV or IPV during the pandemic. Finally, 16 articles were included in this systematic review (Figure 1, for PRISMA flow diagram).

### 3.2 The Characteristics of the Included Studies

The summary of the characteristics of the included studies is listed in Table 2. Of 16 included studies, 2 studies were conducted in Jordan (Abujilban et al. 2022, 2023), 2 studies were in Turkey (Atilla, Yavuz, and Kocaöz 2023; Ozgurluk et al. 2023), 3 studies in Iran (Jalili et al. 2023; Maharlouei et al. 2023; Naghizadeh, Mirghafourvand, and Mohammadirad 2021), 2 studies were in the United States (Avalos et al. 2023; Krishnamurti et al. 2021), four studies in Ethiopia (Asratie 2022; Fetene et al. 2022; Teshome et al. 2021; Wood et al. 2022), 1 study was in China (Wu et al. 2022), 1 study in Brazil (Biagio et al. 2024), and 1 in Canada (Muldoon et al. 2021). The included studies were published between 2021 and 2024. Of 16 studies included, 156,775 pregnant women participated, and the sample sizes varied from 215 to 77,310 individuals. Of the 16 included studies, 14 were cross‐sectional designs (2, 4, 5, 7, 19–27, and 29), and 2 were prospective cohorts (Asratie 2022; Biagio et al. 2024). Regarding the sampling method, in seven studies, the convenience method was used for data collection (Abujilban et al. 2023; Atilla, Yavuz, and Kocaöz 2023; Avalos et al. 2023; Biagio et al. 2024; Krishnamurti et al. 2021; Naghizadeh, Mirghafourvand, and Mohammadirad 2021; Ozgurluk et al. 2023). In three studies, multi‐stage random (Fetene et al. 2022; Jalili et al. 2023; Wu et al. 2022) and in one study, mixed randomized and convenience were used (Maharlouei et al. 2023). Other sampling methods, such as purposive (Wood et al. 2022), snowball (Abujilban et al. 2022), and mixed randomized and convenience (Asratie 2022), were used in three studies. Notably, the sampling method used in two studies was not reported (Muldoon et al. 2021; Teshome et al. 2021). The measurement tools in the included studies were significantly different, whereas most of them used valid questionnaires to assess the prevalence of violence against pregnant women during the COVID‐19 pandemic. Of 16 included studies, 6 studies were conducted in the maternity clinic (Abujilban et al. 2023; Atilla, Yavuz, and Kocaöz 2023; Jalili et al. 2023; Maharlouei et al. 2023; Teshome et al. 2021; Wood et al. 2022), and 8 studies sampling were in the hospital (Asratie 2022; Atilla, Yavuz, and Kocaöz 2023; Avalos et al. 2023; Krishnamurti et al. 2021; Muldoon et al. 2021; Naghizadeh, Mirghafourvand, and Mohammadirad 2021; Ozgurluk et al. 2023; Wu et al. 2022). In addition, one study was carried out through online social media (Abujilban et al. 2022), and one study in the community (house to house) (Fetene et al. 2022).

## 4 Meta‐Analysis Results

### 4.1 Physical Violence

The prevalence of physical violence against pregnant women during COVID‐19 according to the sampling place of the included study is shown in Figure 2a. According to the clinic sampling place, the highest prevalence of physical violence was in the Abujilban study in Jordan, which was reported as 46.10% (95% CI, 39.69%–52.51%), and the lowest prevalence was in the Maharlouei study in Iran which was reported as 7.70% (95% CI, 5.89%–9.51%). On the basis of the combining of the results of five studies conducted in a clinic environment, the total prevalence of physical violence against pregnant women with a CI of 95% and based on the REM was estimated to be 21.17% (95% CI, 6.67%–35.67%). In addition, the prevalence of physical violence according to the hospital sampling environment and another sampling environment, such as online social media and community, was estimated at 3.43% (1.13%–5.72%) and 21.45% (95% CI, 4.99%–37.91%), respectively.

FIGURE 2(a) The prevalence of physical violence according to the sampling place. (b) The forest plot of physical violence according to the quality assessment score.

**Figure:**
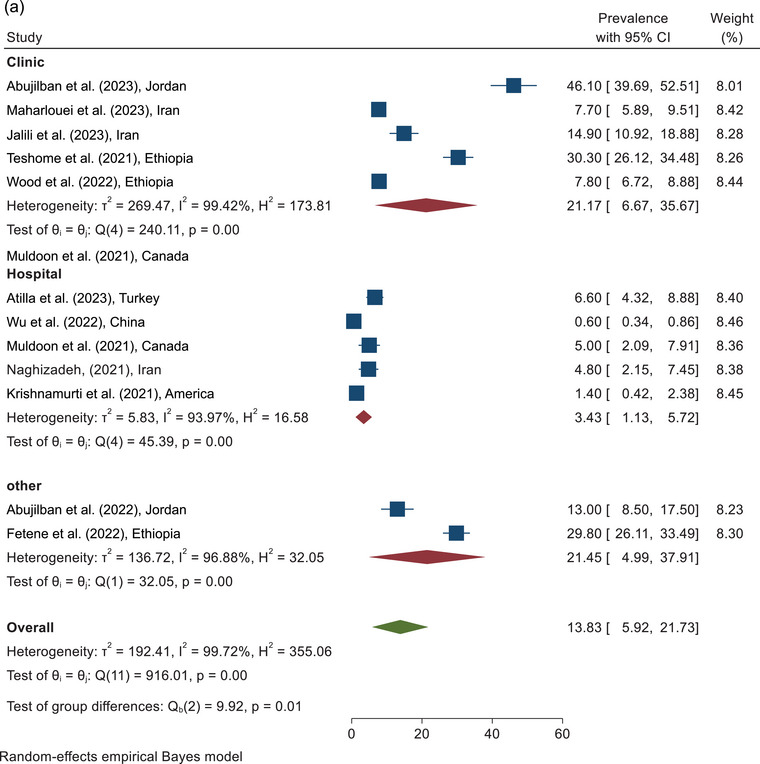

**Figure:**
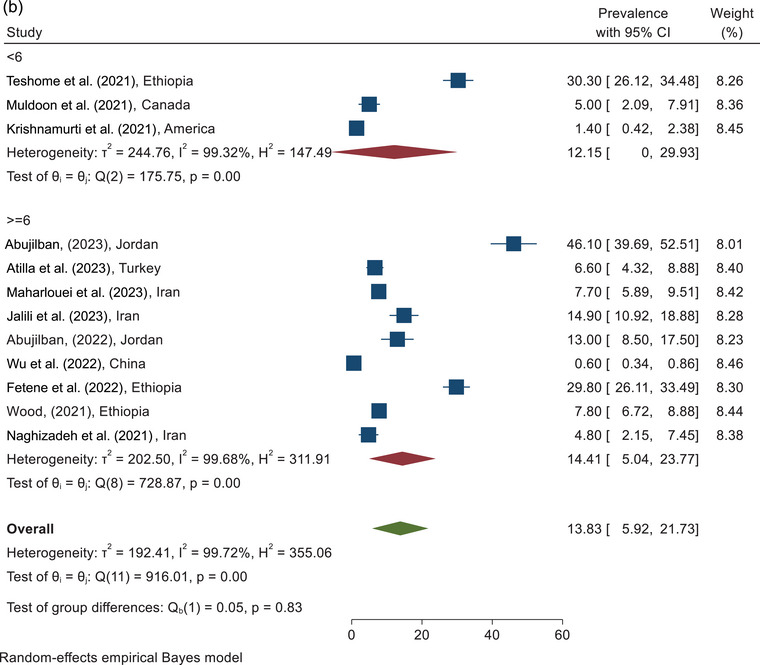

The prevalence of physical violence against pregnant women during COVID‐19, according to the methodological quality of the included study, is shown in Figure 2b. Studies were divided into two categories: <6 and ≥6 (Appendix 1). According to combining the results of three studies analyzed in the category of <6 score, the prevalence of physical violence against pregnant women was estimated as 12.15% (95% CI, 0.0%–29.93%). According to the analysis of the nine studies with a quality score ≥6, the highest prevalence of physical violence was in Abujilban et al., study which was reported 46.10% (95% CI, 39.69%–52.51%), and the lowest prevalence was in Wu et al., study which was estimated 0.60% (95% CI, 0.34%–086%). In addition, the prevalence of physical violence based on the analysis of the nine studies with a quality score ≥6 and based on the REM was reported as 14.41% (95% CI, 5.04%–23.77%). The overall prevalence of physical violence against pregnant women during COVID‐19, according to the combining the results of 12 studies, was estimated at 13.83 (95% CI, 5.92%–21.73%) (Figure 2b).

### 4.2 Psychological Violence

The prevalence of psychological violence against pregnant women during COVID‐19 according to the sampling environment of the included study is shown in Figure 3a. According to combining the results of five studies conducted in maternity clinics, the prevalence of psychological violence against pregnant women was estimated as 64.76% (95% CI, 40.74%–88.78%). The prevalence of psychological violence according to the combining the results of the five studies conducted in hospitals was reported as 15.83% (95% CI, 1.85%–29.80%). According to combining the results of two studies with other sampling places, the prevalence of psychological violence was estimated at 38.32% (95% CI, 15.39%–61.25%) (Figure 3a).

FIGURE 3(a) The forest plot of psychological violence according to the sampling place. (b) The forest plot of psychological violence according to the quality assessment score.

**Figure:**
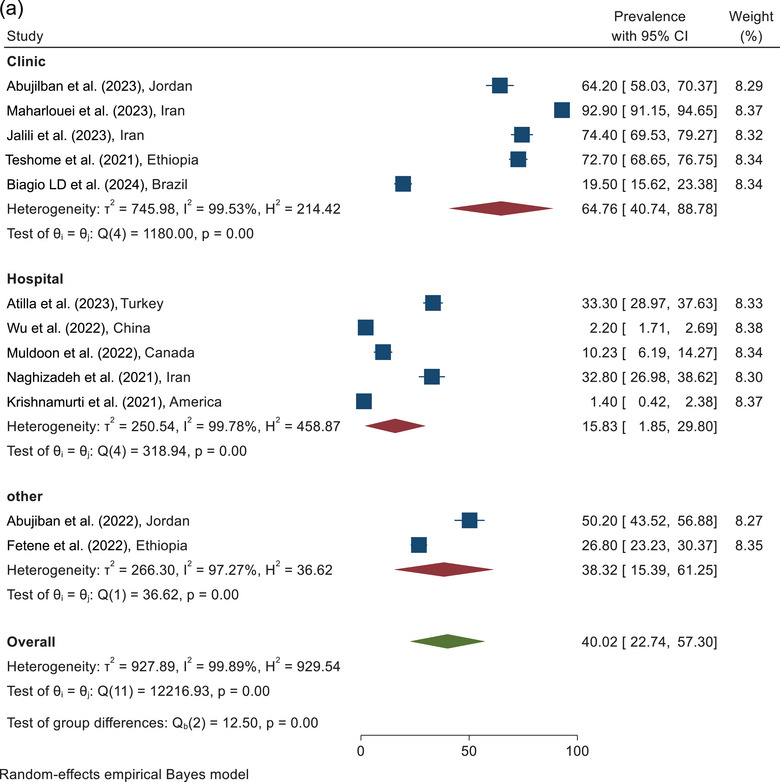

**Figure:**
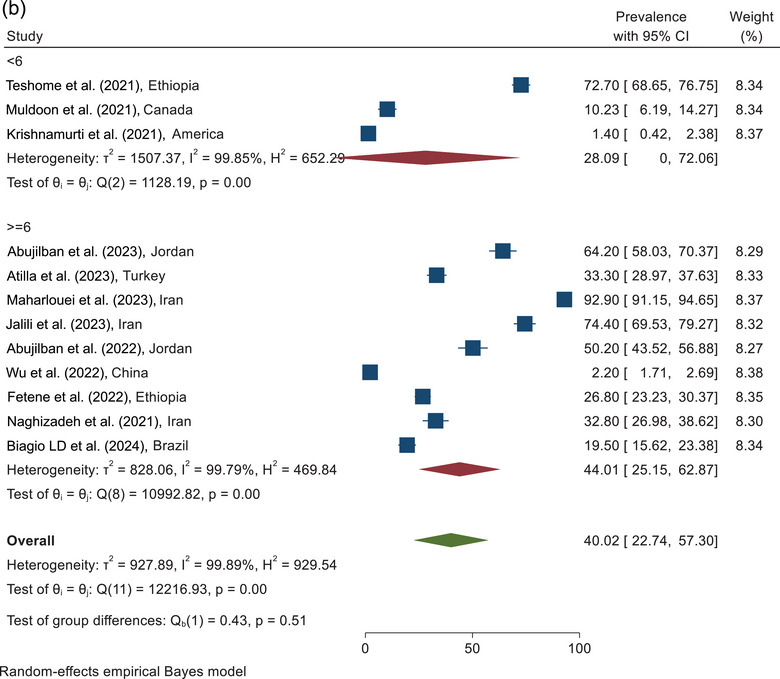

According to combining the results of three studies in the category of <6 score, the prevalence of psychological violence was estimated as 28.09% (95% CI, 0.0%–72.06%). On the basis of the analysis of the nine studies with quality scores≥6, the prevalence of psychological violence was reported as 44.01% (95% CI, 25.15%–62.87%). The overall prevalence of psychological violence against pregnant women during COVID‐19, according to the combining the results of 12 studies, was estimated at 40.02% (95% CI, 22.74%–57.30%) (Figure 3b).

### 4.3 Sexual Violence

The prevalence of sexual violence against pregnant women during COVID‐19 according to the sampling place of the included study is shown in Figure 4a. According to combining the results of five studies conducted in maternity clinics, the prevalence of sexual violence against pregnant women was estimated as 25.34% (95% CI, 9.60%–41.09%). The prevalence of sexual violence according to the combining the results of the five studies conducted in hospitals was reported as 4.13% (95% CI, 0.0%–8.27%). According to combining the results of two studies with other sampling places, the prevalence of sexual violence was reported at 16.78% (95% CI, 6.00%–27.56%) (Figure 4b).

FIGURE 4(a) The forest plot of sexual violence according to the sampling place. (b) The forest plot of sexual violence according to the quality assessment score.

**Figure:**
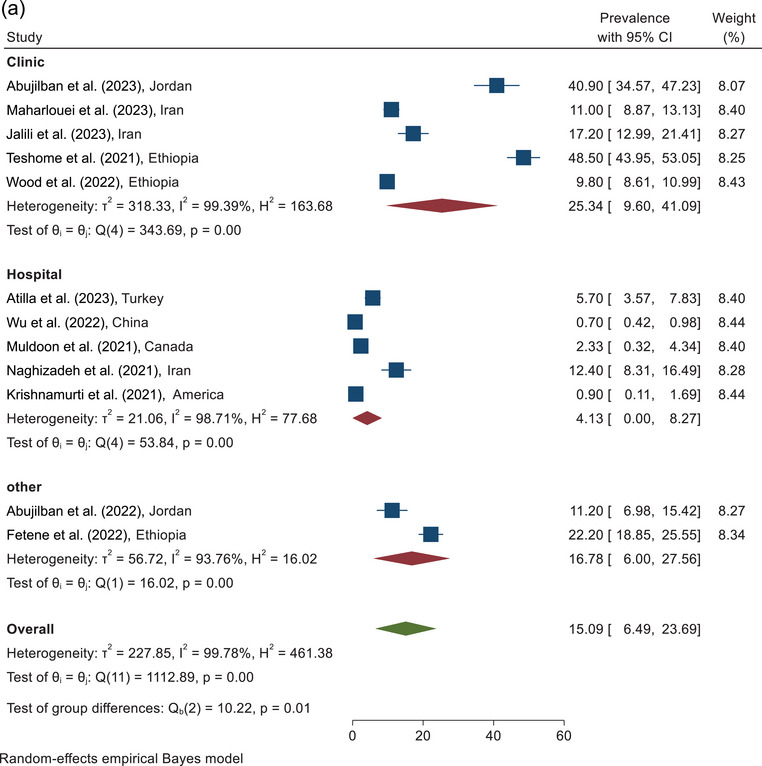

**Figure:**
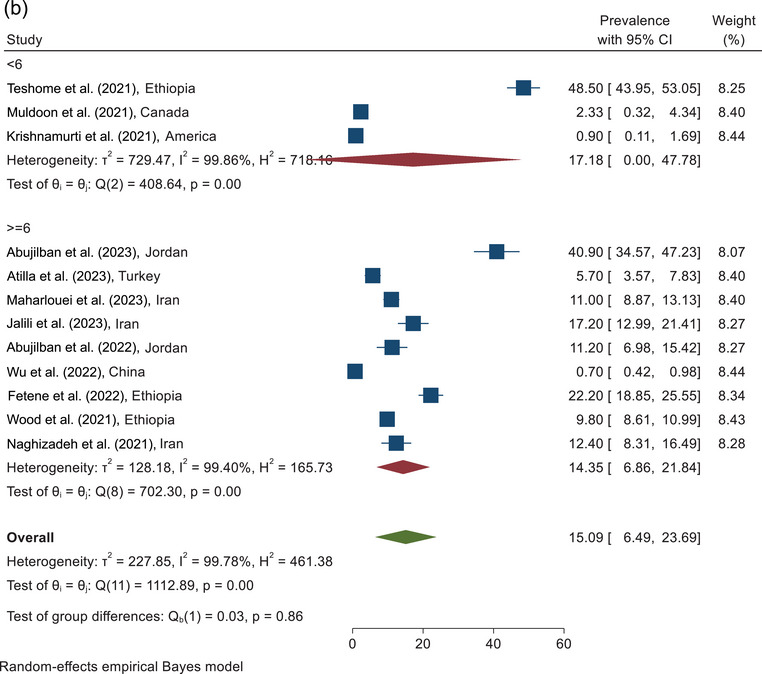

On the basis of the combining the results of the three studies in the category of <6 score, the prevalence of sexual violence was reported as 17.18% (95% CI, 0.0%–47.78%). On the basis of the assessment of the nine studies analyzed with a quality score ≥6, the prevalence of sexual violence was estimated as 14.35% (95% CI, 6.86%–21.84%). The pooled prevalence of sexual violence against pregnant women, according to the combining the results of 12 studies, was estimated at 15.09% (95% CI, 6.49%–23.69%) (Figure 4b).

### 4.4 Total IPV

The prevalence of total IPV violence against pregnant women during COVID‐19 according to the sampling place is shown in Figure 5a. According to the combining the results of six studies conducted in the hospital, the prevalence of total IPV against pregnant women was estimated as 35.30% (95% CI, 18.32%–52.28%). According to the combined results of the four studies conducted in the clinic, the prevalence of total IPV was 30.41% (95% CI, 11.16%–71.97%). According to combining the results of three studies with other sampling places, the prevalence of total IPV was reported to be 43.32% (95% CI, 8.69%–77.96%). The pooled prevalence of the total IPV against pregnant women during COVID‐19, according to the results of 13 studies, was estimated at 35.64% (95% CI, 20.10%–51.19%) (Figure 5a).

FIGURE 5(a) Forest plot of total IPV based on sampling place. (b) Forest plot of total IPV based on quality assessment score. IPV, intimate partner violence.

**Figure:**
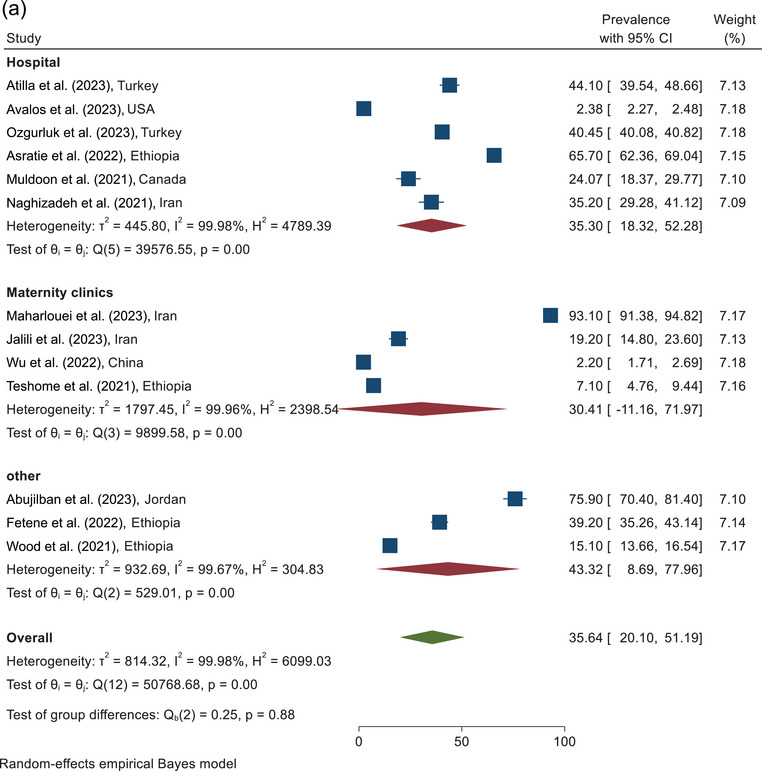

**Figure:**
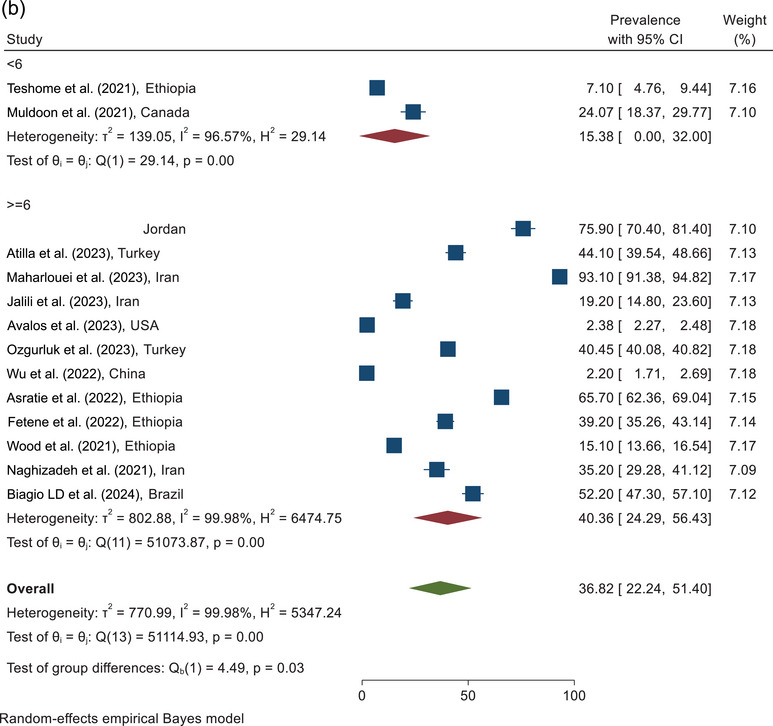

According to combining the results of three studies in the category of <6 score, the prevalence of total IPV was estimated as 15.38% (95% CI, 0.0%–32.00%). On the basis of the analysis of the 12 studies with quality scores≥6, the prevalence of total IPV was reported as 40.36% (95% CI, 24.29%–56.43%). The pooled prevalence of the total IPV against pregnant women during COVID‐19, according to the combined results of 15 studies, was estimated at 36.82% (95% CI, 22.24%–51.40%) (Figure 5b).

### 4.5 The Associated Factors of Each Type of DV or IPV Against Pregnant Women During the COVID‐19 Pandemic, According to the Included Studies

#### 4.5.1 Psychological Violence

Only one study reported the associated factors of psychological violence during the COVID‐19 (Biagio et al. 2024). Factors such as being single/divorced and widowed and the mental health changes based on the GHQ questionnaire were significantly associated with psychological violence.

#### 4.5.2 Physical Violence

According to the results of two included studies, women's lower educational level, weak mutual understanding, lower marriage duration, being house workers, and 30–34 years of age groups were the significant contributing factors of physical violence against pregnant women during the COVID‐19 pandemic (Abujilban et al. 2023; Maharlouei et al. 2023).

#### 4.5.3 Sexual Violence

According to the included studies, no study assessed the risk factors of sexual violence against pregnant women during the COVID‐19 pandemic. Only in one study, father‐wanted pregnancy (OR = 0.20, 95% CI, 0.07%–0.56%) was a protective factor against sexual DV (Maharlouei et al. 2023).

#### 4.5.4 Total DV or IPV

The factors related to DV or IPV against women during COVID‐19 are listed in Table 2. These associated factors were classified into three main categories: sociodemographic, reproduction, interpersonal, psychological, and medical factors.

The sociodemographic and reproduction factors, such as being single, husband's unemployment or being worker, lower women's and their husband's educational status or illiteracy of couples, urban residential place, women's lost job, husband's smoking, economic downturn or household income below the municipal median, no parity or parity three or more, nulliparity, first pregnancy and unintended pregnancy, were significantly associated with DV or IPV against women during the COVID‐19 according to the included studies.

According to the included studies, factors such as moderate‐bad‐very bad marital relationship, an unsafe or unstable living situation, marital conflict, verbal fighting and weak understanding of each other, not being the primary decision maker of family planning or husband decision‐making, and prolonged spouses’ stay at home during COVID were the significant interpersonal factors associated DV or IPV against women during the COVID‐19. Psychological factors such as prenatal anxiety, depression, or postnatal depression were associated significantly with DV or IPV against women during COVID‐19.

According to the results of the included studies, the significant medical‐associated factors of DV or IPV against pregnant women during COVID‐19 were no ambulance service, not having healthcare provider support, and partner alcohol and drug use.

#### 4.5.5 Quality Assessment of the Included Studies

The quality of the two studies was assessed using the NOS for cohort studies, and both had good methodological quality (Asratie 2022; Biagio et al. 2024). Fourteen cross‐sectional studies in this review were methodologically assessed through NOS cross‐sectional studies. Among them, 11 scored 6, 2 studies scored 5 (4 and 5), and only 1 received a 4 score (Krishnamurti et al. 2021). The details of the studies’ scoring are presented in Tables 3 and 4.

## 5 Discussion

This study aimed to systematically assess and analyze the prevalence and associated factors of DV against pregnant women during the COVID‐19 pandemic. In this study, the prevalence of physical, psychological, sexual, and total IPV against pregnant women was analyzed according to two characteristics of sampling place and methodological quality of the included studies and separately reported. Although there are some systematic review and meta‑analysis studies on the prevalence of DV during the COVID‑19 pandemic, there was no systematic review and meta‐analysis regarding the prevalence and associated factors of DV during pregnancy in the period of COVID‐19 pandemic which assessed all published studies until 2024. In the meta‐analysis processes, studies were separately analyzed on the basis of the sampling place, such as clinic and hospital. The results showed that the prevalence of all types of violence in maternity clinics was higher than in hospitals. The higher prevalence in clinics can be due to the higher possibility of assessment during prenatal visits and the existence of screening systems for psychological assessment of pregnant women; therefore, the possibility of reporting to the maternity clinics was higher.

According to the results of this study, the overall prevalence of physical violence against pregnant women, according to combining the results of 12 studies, was estimated at 13.83 (95% CI, 5.92%–21.73%). The results of a systematic review and meta‐analysis study aimed to investigate the IPV against pregnant women during the COVID‐19 pandemic; the prevalence of physical violence was reported at 14% (95% CI, 7.0%–20.0%) (Huldani et al. 2022). Another study with a similar study design showed that 11% of pregnant women had experienced physical violence (95% CI, 6.0%–17%) (Aghababaei et al. 2024). In another systematic review and meta‐analysis, the rate of physical IPV was 18.0% (95% CI 15.1%–20.9%) (Raziani et al. 2024). Although three mentioned studies assessed the prevalence of violence against pregnant women during COVID‐19, this current study investigated all studies until 2024, whereas these mentioned studies' search process covers the studies until 2022 and also had different inclusion and exclusion criteria. According to the results of a study that examined the prevalence of physical violence against pregnant women in non‐COVID‐19 era, the prevalence of physical violence during pregnancy was reported as 10.7% (Akrami 2006). Comparing the results of this study with this current systematic review and meta‐analysis showed that the physical violence against pregnant women during the COVID‐19 was increased.

The results of this study indicated that the pooled prevalence of psychological and sexual violence against pregnant women during COVID‐19, according to the combining the results of 12 studies, was estimated at 40.02% (95% CI, 22.74%–57.30%) and 15.09% (95% CI, 6.49%–23.69%), respectively. According to the results of systematic review and meta‐analysis studies, the prevalence of psychological and sexual violence against pregnant women was reported at 24.0%–43.2% and 6.0%–11.0% (Aghababaei et al. 2024; Huldani et al. 2022; Raziani et al. 2024). The estimated prevalence of psychological violence estimated in this study is approximately similar to the previously published meta‐analysis in this regard. According to the results of a meta‑analysis that assessed the prevalence of DV in non‐COVID‐19 pregnant women in Ethiopia, the overall prevalence of psychological violence was reported to be 27%, which was significantly lower than its prevalence during COVID‐19 assessed in this current study (Bifftu and Guracho 2022). According to the results of two systematic reviews and meta‐analyses, 6.0%–14.0% of pregnant women experienced sexual violence (Aghababaei et al. 2024; Huldani et al. 2022). The results of previous studies were approximately similar to this current study. Regarding sexual violence, in a study that assessed the global prevalence of sexual violence against pregnant women in the non‐COVID‐19 period, the pooled proportion of sexual violence was 0.31 (95% CI: 0.22%–0.40%) (Shen et al. 2022) which was significantly lower than COVID‐19 pandemic. Overall, sexual violence against pregnant women is a major public health challenge and needs serious attention.

In this study, the total prevalence of the total IPV against pregnant women during COVID‐19 was estimated at 36.82% (95% CI, 22.24%–51.40%). According to the results of three systematic reviews and meta‐analyses, the prevalence of IPV against pregnant women was reported at 23%–51.5% (Aghababaei et al. 2024; Huldani et al. 2022; Raziani et al. 2024). The prevalence of IPV among pregnant women during the non‐COVID‐19 pandemic was estimated at 5.5%–26.1% (Alebel et al. 2018; Román‐Gálvez et al. 2021). Comparing the results of these studies with this current study showed that the prevalence of total IPV increased during the COVID‐19 pandemic. Overall during the COVID‐19 pandemic, in some countries, increased reports of violence against women to shelters and police are observed, whereas others reported reductions, in part due to physical distancing, inaccessibility of or reductions in service availability and social support for abusive women (Ain et al. 2023; Biagio et al. 2024).

The results of this study showed that factors such as sociodemographic, reproduction, interpersonal, psychological, and medical were associated with total IPV among pregnant women. The results of a systematic review that examined the associated factors of DV against pregnant women during the pandemic era showed that factors such as lockdown and quarantine, economic strains and reduced incomes, unintended pregnancies, and limited access to healthcare services for antenatal care were the leading causes of increased each type of DV against pregnant women (Savvoudi et al. 2024). In another systematic review, factors such as partner alcohol use, income constraints during the COVID‐19 pandemic, partner's literacy level, and decision‐making in the household were the significant associated of IPV against pregnant women during COVID‐19, which were consistent with this current study's results (Alemu et al. 2024).

## 6 Limitations

DV underreporting is a common issue, especially during crises and humanitarian emergencies and the prevalence reported by single studies may be affected by this issue. The lack of reporting odds ratio of the associated factors of DV against pregnant women caused us only to systematically report these factors, and we will not be able to meta‐analysis them. In addition, language and selection bias and any other biases, like sample size, lack of prior research studies, and some other databases missing Cochrane Central were other limitations of this study.

## 7 Conclusion

The systematic review showed that psychological, physical, and sexual violence were the most common forms of violence assessed during the COVID‐19 pandemic. Although the prevalence of all types of violence against pregnant women increased during the COVID‐19 pandemic compared to other times, the results of the present study indicated that psychological violence was the most common violence reported by pregnant women. In contrast, the prevalence of physical violence is lower than other types of violence reported in pregnant women during the COVID‐19 pandemic. Given the considerable effect of violence on pregnant women's health status, it is necessary to identify high‑risk women as the victims of violence and consider and design preventive interventions in susceptible pregnant women worldwide. The results of this study clarify the importance of attention to issues such as DV as an emergency event during the pandemic and other crises. As pregnant women need special protection in this period and each unfavorable event can be associated with adverse outcomes, the results of this study highlighted the higher susceptibility of pregnant women during pregnancy and emphasized the importance of considering the appropriate interventions to improve the psychological health of pregnant women and also designing the considerable intervention to reduce the prevalence of DV or IPV among pregnant women. In addition, the results of this study highlighted the considerable role of healthcare providers such as midwives, gynecologists, psychologists, and psychiatrics in clinical and psychological assessments of pregnant women during pregnancy especially during the pandemic.

By raising the awareness and knowledge of pregnant women regarding the importance of reporting each type of violence during pregnancy and informing them about secure and safe spaces, introducing the resources to cope with and escape abusive situations, and providing general and special supportive care in vulnerable cases are among the important public policies to reduce the possible antenatal and perinatal adverse outcomes among pregnant women.

## Author Contributions

**Kosar Miraei Mohammadi**: data curation, methodology, writing ‐ original draft. **Zohreh Shahhosseini**: conceptualization, methodology, validation, supervision. **Mahtab Haji Mohammadreza**: data curation, investigation. **Fatemeh Heshmatnia**: writing ‐ original draft, investigation. **Roya Nikbakht**: formal analysis, software, methodology, validation. **Erfan Ghasemi**: software. **Maryam Jafari**: writing ‐ review and editing. **Hamed Milani**: writing ‐ review and editing, data curation, conceptualization. **Marzieh Azizi**: conceptualization, writing ‐ original draft, writing ‐ review and editing, project administration, supervision, methodology, data curation, investigation.

## Ethics Statement

The authors have nothing to report.

## Consent

The authors have nothing to report.

## Conflicts of Interest

The authors declare no conflicts of interest.

### Peer Review

The peer review history for this article is available at https://publons.com/publon/10.1002/brb3.70345

## Data Availability

The datasets used and/or analyzed during the current study are available from the corresponding author upon reasonable request.

## Appendix I

### I.1

The analysis of the included studies according to the sampling place and quality assessment score separately sorted by the type of violence.

**Table:**

**Outcome**	**Variable**	***N* of studies**	**Combined prevalence (95% CI)**	**Heterogeneity**		**Meta‐regression**	
				***I* ^2^ (%)**	***p* value**	**Coefficient**	***p* value**
**Physical**	**Sampling place**						
	Clinic	5	21.17 (6.67, 35.67)	99.42	<0.001	Reference	
	Hospital	5	3.43 (1.13, 5.72)	93.97	<0.001	17.32	0.020
	Other	2	21.45 (4.99, 5.72)	96.88	<0.001	17.78	0.071
	**Quality assessment score**						
	<6	3	12.15 (0, 29.93)	99.32	<0.001	Reference	
	≥6	9	14.41 (5.04, 23.77)	99.68	<0.001	2.28	0.815
	**Total**	12	13.83 (5.92, 21.73)	99.72	<0.001	—	
**Psychological**	**Sampling place**						
	Clinic	5	64.76 (40.74, 88.78)	99.53	<0.001	Reference	
	Hospital	5	15.83 (1.85, 29.80)	99.78	<0.001	48.87	<0.001
	Other	2	38.32 (15.39, 61.25)	97.27	<0.001	22.50	0.219
	**Quality assessment score**						
	<6	3	28.09 (0, 72.06)	99.85	<0.001	Reference	
	≥6	9	44.01 (25.15, 62.87)	99.79	<0.001	15.28	0.442
	**Total**	12	40.02 (22.74, 57.30)	99.89	<0.001	—	
**Sexual**	**Sampling place**						
	Clinic	5	25.34 (9.60, 41.09)	99.39	<0.001	Reference	
	Hospital	5	4.13 (0, 8.27)	98.71	<0.001	20.84	0.009
	Other	2	16.78 (6.00, 27.56)	93.76	<0.001	12.36	0.242
	**Quality assessment score**						
	<6		17.18 (0, 47.78)	99.86	<0.001	Reference	
	≥6		14.35 (6.86, 21.84)	99.40	<0.001	−2.60	0.806
	**Total**		15.09 (6.49, 23.69)	99.78	<0.001	—	
**Total IPV**	**Sampling place**						
	Clinic	6	43.75 (15.31, 72.18)	99.87	<0.001	Reference	
	Hospital	6	30.54 (13.46, 47.62)	99.99	<0.001	13.18	0.420
	Other	1	39.20 (35.26, 43.14)	—	—	8.64	0.783
	**Quality assessment score**						
	<6	2	15.38 (0, 32)	96.57	<0.001	Reference	
	≥6	9	40.36 (24.25, 56.43)	99.98	<0.001	24.81	0.236
	**Total**	12	36.82 (22.24, 51.40)	99.98	<0.001	—	

## References

[brb370345-bib-0002] Abujilban, S. , A. AbuAbed , L. Mrayan , et al. 2023. “Pregnant Women's Experiences With Intimate Partner Violence One Year After the COVID‐19 Pandemic in Jordan.” Nursing Open 10, no. 7: 4286–4297. 36826391 10.1002/nop2.1669 PMC10277416

[brb370345-bib-0003] Abujilban, S. , L. Mrayan , S. Hamaideh , S. Obeisat , and J. Damra . 2022. “Intimate Partner Violence Against Pregnant Jordanian Women at the Time of COVID‐19 Pandemic's Quarantine.” Journal of Interpersonal Violence 37, no. 5–6: NP2442–NP2464. 33403908 10.1177/0886260520984259

[brb370345-bib-0004] Aghababaei, S. , Z. Masoumi , R. Tahmasebi , E. Jenabi , Z. Toosi , and S. Ghelichkhani . 2024. “Violence Against Women During Pregnancy and Its Dimensions in COVID‐19 Pandemic: A Systematic Review and Meta‐Analysis.” Industrial Psychiatry Journal 33, no. S1: S8–S18. 39534138 10.4103/ipj.ipj_167_23 PMC11553599

[brb370345-bib-0005] Ain, Q. U. , C. Ozkaya , A. Amin , et al. 2023. “Violence Against Women During the Covid‐19 Pandemic: Scoping Review of the Literature in Collaboration With the World Health Organization Protocol.” International Journal of Educational Research Open 5: 100267.

[brb370345-bib-0006] Akrami, M. N. Z 2006. “Prevalence of Physical Violence Against Pregnant Women and Effects on Maternal and Birth Outcomes.” Acta Medica Iranica 44: 95–100.

[brb370345-bib-0007] Alebel, A. , G. D. Kibret , F. Wagnew , et al. 2018. “Intimate Partner Violence and Associated Factors Among Pregnant Women in Ethiopia: A Systematic Review and Meta‐Analysis.” Reproductive Health 15: 1–12. 30514311 10.1186/s12978-018-0637-x PMC6278116

[brb370345-bib-0008] Alemu, T. G. , T. T. Tamir , B. S. Workneh , et al. 2024. “Intimate Partner Violence and Associated Factors Among Women During the COVID‐19 Pandemic in Ethiopia: A Systematic Review and Meta‐Analysis.” Frontiers in Global Women's Health 5: 1425176. 10.3389/fgwh.2024.1425176 PMC11377230 39246731

[brb370345-bib-0009] Asratie, M. H. 2022. “Domestic Violence During COVID‐19 Pandemic Among Pregnant Women Registered for Antenatal Care and Selected Adverse Pregnancy Outcomes in Amhara Region Ethiopia: Prospective Cohort Study Design.” Clinical Epidemiology and Global Health 17: 101146. 36128548 10.1016/j.cegh.2022.101146 PMC9479382

[brb370345-bib-0010] Atilla, R. , A. Yavuz , and S. Kocaöz . 2023. “Exposure of Pregnant Women to Intimate Partner Violence During the Pandemic in Turkey and Influencing Factors.” Journal of Community Health Nursing 40, no. 1: 1–13. 36602774 10.1080/07370016.2022.2094708

[brb370345-bib-0011] Avalos, L. A. , G. T. Ray , S. E. Alexeeff , et al. 2023. “Association of the COVID‐19 Pandemic With Unstable and/or Unsafe Living Situations and Intimate Partner Violence Among Pregnant Individuals.” JAMA Network Open 6, no. 2: e230172. 36811863 10.1001/jamanetworkopen.2023.0172 PMC9947729

[brb370345-bib-0012] Belay, S. , A. Astatkie , M. Emmelin , and S. G. Hinderaker . 2019. “Intimate Partner Violence and Maternal Depression During Pregnancy: A Community‐Based Cross‐Sectional Study in Ethiopia.” PLoS ONE 14, no. 7: e0220003. 31365566 10.1371/journal.pone.0220003 PMC6668805

[brb370345-bib-0013] Biagio, L. D. , D. Devakumar , L. F. de Carvalho , et al. 2024. “Factors Associated With Domestic Violence in Pregnant Women During the COVID‐19 Pandemic: Araraquara Cohort Study.” BJPsych Bulletin 17: 1–7. 10.1192/bjb.2024.43 PMC12314416 38757198

[brb370345-bib-0014] Bifftu, B. B. , and Y. D. Guracho . 2022. “Determinants of Intimate Partner Violence Against Pregnant Women in Ethiopia: A Systematic Review and Meta‐Analysis.” BioMed Research International 2022, no. 1: 4641343. 35378786 10.1155/2022/4641343 PMC8976645

[brb370345-bib-0015] Boxall, H. , A. Morgan , and R. Brown . 2020. “The Prevalence of Domestic Violence Among Women During the COVID‐19 Pandemic.” Australasian Policing 12, no. 3: 38–46.

[brb370345-bib-0016] Brownridge, D. A. , T. L. Taillieu , K. A. Tyler , A. Tiwari , K. L. Chan , and S. C. Santos . 2011. “Pregnancy and Intimate Partner Violence: Risk Factors, Severity, and Health Effects.” Violence Against Women 17, no. 7: 858–881. 21775311 10.1177/1077801211412547

[brb370345-bib-0017] Every‐Palmer, S. , M. Jenkins , P. Gendall , et al. 2020. “Psychological Distress, Anxiety, Family Violence, Suicidality, and Wellbeing in New Zealand During the COVID‐19 Lockdown: A Cross‐Sectional Study.” PLoS ONE 15, no. 11: e0241658. 33147259 10.1371/journal.pone.0241658 PMC7641386

[brb370345-bib-0018] Fetene, G. , M. S. Alie , D. Girma , and Y. Negesse . 2022. “Prevalence and Its Predictors of Intimate Partner Violence Against Pregnant Women Amid COVID‐19 Pandemic in Southwest Ethiopia, 2021: A Cross‐Sectional Study.” SAGE Open Medicine 10: 20503121221079317. 35223032 10.1177/20503121221079317 PMC8873970

[brb370345-bib-0019] Garg, S. , R. Rustagi , M. M. Singh , and K. Engtipi . 2020. “Effect of Intimate Partner Violence on Maternal and Birth Outcomes of Pregnancy Among Antenatal Clinic Attendees in Delhi: A Prospective Observational Study.” Indian Journal of Community Medicine 45, no. 4: 501–505. 33623210 10.4103/ijcm.IJCM_538_19 PMC7877408

[brb370345-bib-0020] Graham‐Harrison, E. , A. Giuffrida , H. Smith , and L. Ford . 2020. “Lockdowns Around the World Bring Rise in Domestic Violence.” The Guardian 28: 2020.

[brb370345-bib-0021] Heaman, M. I. , P. J. Martens , M. D. Brownell , M. J. Chartier , S. A. Derksen , and M. E. Helewa . 2019. “The Association of Inadequate and Intensive Prenatal Care With Maternal, Fetal, and Infant Outcomes: A Population‐Based Study in Manitoba, Canada.” Journal of Obstetrics and Gynaecology Canada 41, no. 7: 947–959. 30639165 10.1016/j.jogc.2018.09.006

[brb370345-bib-0022] Huldani, H. , W. Kamal Abdelbasset , S. Abdalkareem Jasim , et al. 2022. “Intimate Partner Violence Against Pregnant Women During the COVID‐19 Pandemic: A Systematic Review and Meta‐Analysis.” Women & Health 62, no. 6: 556–564. 35791678 10.1080/03630242.2022.2096755

[brb370345-bib-0023] Islam, M. J. , L. Broidy , K. Baird , and P. Mazerolle . 2017. “Intimate Partner Violence Around the Time of Pregnancy and Postpartum Depression: The Experience of Women of Bangladesh.” PLoS ONE 12, no. 5: e0176211. 28472056 10.1371/journal.pone.0176211 PMC5417480

[brb370345-bib-0024] Jalili, M. , S. Kohan , M. J. Tarrahi , and F. Torabi . 2023. “Domestic Violence During Pregnancy and Its Predictive Factors During the COVID‐19 Epidemic Among Primiparous Women in Isfahan.” Journal of Hayat 29: 77–88.

[brb370345-bib-0025] Krishnamurti, T. , A. L. Davis , B. Quinn , A. F. Castillo , K. L. Martin , and H. N. Simhan . 2021. “Mobile Remote Monitoring of Intimate Partner Violence Among Pregnant Patients During the COVID‐19 Shelter‐in‐Place Order: Quality Improvement Pilot Study.” Journal of Medical Internet Research 23, no. 2: e22790. 33605898 10.2196/22790 PMC7899202

[brb370345-bib-0026] Loiseau, M. , J. Cottenet , S. Bechraoui‐Quantin , et al. 2021. “Physical Abuse of Young Children During the COVID‐19 Pandemic: Alarming Increase in the Relative Frequency of Hospitalizations During the Lockdown Period.” Child Abuse & Neglect 122: 105299. 34488053 10.1016/j.chiabu.2021.105299 PMC8435815

[brb370345-bib-0027] Luchini, C. , B. Stubbs , M. Solmi , and N. Veronese . 2017. “Assessing the Quality of Studies in Meta‐Analyses: Advantages and Limitations of the Newcastle Ottawa Scale.” World Journal of Meta‐Analysis 5, no. 4: 80–84.

[brb370345-bib-0028] Maharlouei, N. , S. Roozmeh , Z. Roozegar , et al. 2023. “Intimate Partner Violence During Pregnancy in COVID‐19 Pandemic: A Cross‐Sectional Study From South‐West of Iran.” BMC Public Health [Electronic Resource] 23, no. 1: 325. 36788571 10.1186/s12889-023-15258-x PMC9926421

[brb370345-bib-0029] Margulis, A. V. , M. Pladevall , N. Riera‐Guardia , et al. 2014. “Quality Assessment of Observational Studies in a Drug‐Safety Systematic Review, Comparison of Two Tools: The Newcastle–Ottawa Scale and the RTI Item Bank.” Clinical Epidemiology 6: 359. 25336990 10.2147/CLEP.S66677 PMC4199858

[brb370345-bib-0030] Meaney, S. , S. Leitao , E. K. Olander , J. Pope , and K. Matvienko‐Sikar . 2022. “The Impact of COVID‐19 on Pregnant Womens' Experiences and Perceptions of Antenatal Maternity Care, Social Support, and Stress‐Reduction Strategies.” Women and Birth 35, no. 3: 307–316. 33994134 10.1016/j.wombi.2021.04.013 PMC9051126

[brb370345-bib-0031] Muldoon, K. A. , K. M. Denize , R. Talarico , et al. 2021. “COVID‐19 and Perinatal Intimate Partner Violence: A Cross‐Sectional Survey of Pregnant and Postpartum Individuals in the Early Stages of the COVID‐19 Pandemic.” BMJ Open 11, no. 5: e049295. 10.1136/bmjopen-2021-049295 PMC8728372 34045216

[brb370345-bib-0032] Naghizadeh, S. , M. Mirghafourvand , and R. Mohammadirad . 2021. “Domestic Violence and Its Relationship With Quality of Life in Pregnant Women During the Outbreak of COVID‐19 Disease.” BMC Pregnancy and Childbirth 21: 1–10. 33509103 10.1186/s12884-021-03579-x PMC7840794

[brb370345-bib-0033] Ozgurluk, I. , B. Tastekin , S. Yazkan Hira , et al. 2023. “Assessment of the COVID‐19 Pandemic's Impact on Physical Intimate Partner Violence Against Pregnant Women in Ankara (Turkey): A Hospital‐Based Study.” International Journal of Women's Health 15: 1161–1169. 10.2147/IJWH.S419014 PMC10378456 37520182

[brb370345-bib-0034] Page, M. J. , J. E. McKenzie , P. M. Bossuyt , et al. 2021. “The PRISMA 2020 Statement: An Updated Guideline for Reporting Systematic Reviews.” BMJ 372: n71. 33782057 10.1136/bmj.n71 PMC8005924

[brb370345-bib-0035] Piquero, A. R. , W. G. Jennings , E. Jemison , C. Kaukinen , and F. M. Knaul . 2021. “Domestic Violence During the COVID‐19 Pandemic‐Evidence From a Systematic Review and Meta‐Analysis.” Journal of Criminal Justice 74: 101806. 36281275 10.1016/j.jcrimjus.2021.101806 PMC9582712

[brb370345-bib-0036] Rahman, R. , C. Huysman , A. M. Ross , and E. R. Boskey . 2022. “Intimate Partner Violence and the COVID‐19 Pandemic.” Pediatrics 149, no. 6: e2021055792. 35314862 10.1542/peds.2021-055792

[brb370345-bib-0037] Raziani, Y. , L. Hasheminasab , R. G. Gheshlagh , P. Dalvand , V. Baghi , and M. Aslani . 2024. “The Prevalence of Intimate Partner Violence Among Iranian Pregnant Women: A Systematic Review and Meta‐Analysis.” Scandinavian Journal of Public Health 52, no. 1: 108–118. 36207824 10.1177/14034948221119641

[brb370345-bib-0038] Román‐Gálvez, R. M. , S. Martín‐Peláez , B. M. Fernández‐Félix , J. Zamora , K. S. Khan , and A. Bueno‐Cavanillas . 2021. “Worldwide Prevalence of Intimate Partner Violence in Pregnancy. A Systematic Review and Meta‐Analysis.” Frontiers in Public Health 9: 738459. 34527656 10.3389/fpubh.2021.738459 PMC8435609

[brb370345-bib-0039] Savvoudi, D.‐M. , E. Orovou , M. Dagla , G. Kirkou , G. Iatrakis , and E. Antoniou . 2024. “Domestic Violence in Pregnancy During the Pandemic Era: A Systematic Review.” Maedica 19, no. 2: 400. 39188836 10.26574/maedica.2024.19.2.400 PMC11345045

[brb370345-bib-0040] Shen, X. , H. Dong , H. Jiang , et al. 2022. “The Global Prevalence of Sexual Violence Against Pregnant Women: A Systematic Review and Meta‐Analysis.” Women & Health 62, no. 1: 37–45. 34886757 10.1080/03630242.2021.2011824

[brb370345-bib-0041] Sifat, R. I 2020. “Sexual Violence Against Women in Bangladesh During the COVID‐19 Pandemic.” Asian Journal of Psychiatry 54: 102455. 33271734 10.1016/j.ajp.2020.102455 PMC7577916

[brb370345-bib-0042] Stang, A 2010. “Critical Evaluation of the Newcastle‐Ottawa Scale for the Assessment of the Quality of Nonrandomized Studies in Meta‐Analyses.” European Journal of Epidemiology 25, no. 9: 603–605. 20652370 10.1007/s10654-010-9491-z

[brb370345-bib-0043] Teshome, A. , W. Gudu , D. Bekele , M. Asfaw , R. Enyew , and S. D. Compton . 2021. “Intimate Partner Violence Among Prenatal Care Attendees Amidst the COVID‐19 Crisis: The Incidence in Ethiopia.” International Journal of Gynecology & Obstetrics 153, no. 1: 45–50. 33368273 10.1002/ijgo.13566 PMC9087760

[brb370345-bib-0044] Wells, G. , B. Shea , D. O'connell , et al. 2016. “The Newcastle‐Ottawa Scale (NOS) for Assessing the Quality of Nonrandomised Studies in Meta‐Analyses.” Clinical Epidemiology. Ottawa Hospital Research Institute.

[brb370345-bib-0045] Wells, G. A. , B. Shea , D. O'Connell , et al. 2000. The Newcastle‐Ottawa Scale (NOS) for Assessing the Quality of Nonrandomised Studies in Meta‐Analyses. Oxford.

[brb370345-bib-0046] Wilson, A. N. , C. Ravaldi , M. J. Scoullar , et al. 2021. “Caring for the Carers: Ensuring the Provision of Quality Maternity Care During a Global Pandemic.” Women and Birth 34, no. 3: 206–209. 32276778 10.1016/j.wombi.2020.03.011 PMC7141547

[brb370345-bib-0047] Wood, S. , R. Yirgu , A. Wondimagegnehu , J. Qian , R. Milkovich , and M. Decker . 2022. “Impact of the COVID‐19 Pandemic on Intimate Partner Violence During Pregnancy: Evidence From a Multimethods Study of Recently Pregnant Women in Ethiopia.” BMJ Open 12, no. 4: e055790. 10.1136/bmjopen-2021-055790 PMC9006189 35414554

[brb370345-bib-0048] Wu, F. , L. Zhou , C. Chen , et al. 2022. “Association Between Intimate Partner Violence and Prenatal Anxiety and Depression in Pregnant Women: A Cross‐Sectional Survey During the COVID‐19 Epidemic in Shenzhen, China.” BMJ Open 12, no. 5: e055333. 10.1136/bmjopen-2021-055333 PMC9121109 35589360

